# Prescribing Optimal Nutrition and Physical Activity as “First-Line” Interventions for Best Practice Management of Chronic Low-Grade Inflammation Associated with Osteoarthritis: Evidence Synthesis

**DOI:** 10.1155/2012/560634

**Published:** 2012-12-31

**Authors:** Elizabeth Dean, Rasmus Gormsen Hansen

**Affiliations:** ^1^Department of Physical Therapy, Faculty of Medicine, University of British Columbia, Vancouver, BC, Canada V6T 1Z3; ^2^Department of Physical Therapy, Ringsted and Slagelse Hospitals, Region Zealand, Denmark

## Abstract

Low-grade inflammation and oxidative stress underlie chronic osteoarthritis. Although best-practice guidelines for osteoarthritis emphasize self-management including weight control and exercise, the role of lifestyle behavior change to address chronic low-grade inflammation has not been a focus of first-line management. This paper synthesizes the literature that supports the idea in which the Western diet and inactivity are proinflammatory, whereas a plant-based diet and activity are anti-inflammatory, and that low-grade inflammation and oxidative stress underlying osteoarthritis often coexist with lifestyle-related risk factors and conditions. We provide evidence-informed recommendations on how lifestyle behavior change can be integrated into “first-line” osteoarthritis management through teamwork and targeted evidence-based interventions. Healthy living can be exploited to reduce inflammation, oxidative stress, and related pain and disability and improve patients' overall health. This approach aligns with evidence-based best practice and holds the promise of eliminating or reducing chronic low-grade inflammation, attenuating disease progression, reducing weight, maximizing health by minimizing a patient's risk or manifestations of other lifestyle-related conditions hallmarked by chronic low-grade inflammation, and reducing the need for medications and surgery. This approach provides an informed cost effective basis for prevention, potential reversal, and management of signs and symptoms of chronic osteoarthritis and has implications for research paradigms in osteoarthritis.

## 1. Introduction

Best practice guidelines for chronic osteoarthritis focus on self-management, that is, weight control and physical activity, and on pharmacological support for inflammation and pain [1–5]. Despite such guidelines, authorities in the field report a lack of efficacy of current treatments and associated adverse effects [6], with some proposing even greater attention to self-management [7]. Further, although low-grade inflammation underlies chronic osteoarthritis comparable to other conditions with significant lifestyle-related components often presenting concurrently with osteoarthritis, this inflammation has not been a focus of best practice guidelines, particularly of its nonpharmacologic management.

To establish the prescription of optimal nutrition and physical activity as ‘‘first-line” interventions for low-grade inflammation associated with chronic osteoarthritis, we have synthesized three primary lines of support: (1) the literature that supports that the western diet and inactive lifestyle are proinflammatory, and a plant-based diet and regular physical activity are anti-inflammatory; (2) the literature supporting that low-grade inflammation is common across lifestyle-related conditions including osteoarthritis; and (3) evidence-informed recommendations for effecting lifestyle behavior change that can be readily integrated by health practitioners into ‘‘first-line” management. We conclude with implications for clinical practice and research with respect to its paradigm and avenues for future investigation.

## 2. Low-Grade Inflammation and Lifestyle

Human lifestyles have changed dramatically over millennia. With technological and economic advancements in western countries particularly over the past 60 years, lifestyle-related conditions are the leading causes of premature death [13]. With globalization, western diets coupled with inactivity have contributed largely to lifestyle-related conditions which are increasingly prevalent in middle- and low-income countries [14]. Some authorities have not only argued that western diets have contributed to poor chronic health outcomes, but that national food guidelines such as those in the United States have legitimized poor nutrition for several decades further contributing to the pandemic of lifestyle-related conditions [15]. In particular, poor nutritional quality has been reported to contribute to obesity [16], a primary risk factor for osteoarthritis [17], in addition to calorie density.

The factors associated with the typical western lifestyle that impact people's health have been elucidated by cross-cultural studies including seminal work related to Mediterranean diet and exercise patterns and Asian lifestyles. The Mediterranean diet known to be health protective is largely plant based, favors olive oil over animal fats, and is high in fiber, vegetables, and fruits [18]. The China study [19–22] is a prime example. This comprehensive series of studies has shown the serious health consequences of high consumption of meat, dairy, fat, and refined grains and sugar (proinflammatory), and low consumption of whole grains, vegetables and fruits, and legumes and pulses (anti-inflammatory). This unnatural diet for humans contributes to low-grade systemic inflammation and oxidative tissue stress and irritation, placing the immune system in an overactive state, a common denominator of conditions with lifestyle components including arthritis [15]. Both high carbohydrate and high fat consumption contribute to inflammatory and oxidative stress even in healthy people [23]. This effect could accentuate inflammatory conditions such as lowering the threshold for local inflammation in arthritis. Diet-induced weight loss in people who are overweight reduces chronic low-grade inflammation as evidenced by signification reduced C-reactive protein, an inflammation biomarker [24].

In addition, sedentary living and inactivity are hallmarks of western culture. Evidence supports that inactivity is proinflammatory and augments oxidative stress [25], whereas activity when not excessive is anti-inflammatory [26, 27]. More commonly understood about exercise, however, is that inactivity weakens muscles and contributes to joint stress, in addition to reducing stimulation of synovial fluid which cushions the joints and protects the joint spaces [28]. Activity and exercise continue to be primarily recommended and prescribed to people with arthritis to offset these adverse effects. The anti-inflammatory effects of exercise, however, have been well established, and that for maximal anti-inflammatory benefit, broad-based training needs to include resistance and aerobic training [26, 27, 29]. Exercise induced analgesia [30] and stiffness associated with osteoarthritis may reflect both its anti-inflammatory and mechanical effects; however, exercise's anti-inflammatory effects are not discussed in established practice guidelines [1–5]. In sum, the western lifestyle is inherently unhealthy, and lifestyles with nonwestern diets and greater activity levels are typically associated with better health outcomes, for example, traditional Asian and Mediterranean lifestyles [18, 31].

Other lifestyle traits common in western culture are also known to be proinflammatory. Smoking, for example, remains prevalent despite some success in recent decades in reducing its prevalence through public health campaigns. The chronic low-grade inflammation associated with smoking [32, 33] has been linked with inflammatory states associated with ischemic heart disease [34], rheumatoid arthritis [35], and osteoarthritis [36]. Low-grade inflammation has been associated with chronic sleep deprivation and stress [37–40] which are also common in western cultures. Given the well-documented link between low-grade inflammation and oxidative stress, and sleep deprivation and stress [41], a case can be made for assessing and addressing these in the initial assessment and in first-line management of chronic osteoarthritis. In addition, sleep deprivation and stress are common arthritic complaints secondary to discomfort and pain, lending further support for assessing sleep and stress in people with chronic osteoarthritis and intervening as indicated.

Thus, prescribing healthy living strategies in general as well as optimal nutrition (of which weight loss is an additional benefit) and regular physical activity are warranted as being first-line interventions in clinical practice guidelines for conditions such as osteoarthritis associated with chronic low-grade inflammation. These conditions are described in the next section and often coexist as comorbidities in people with osteoarthritis.

## 3. Low-Grade Inflammation and Lifestyle-Related Conditions Including Osteoarthritis

**Figure 1:**
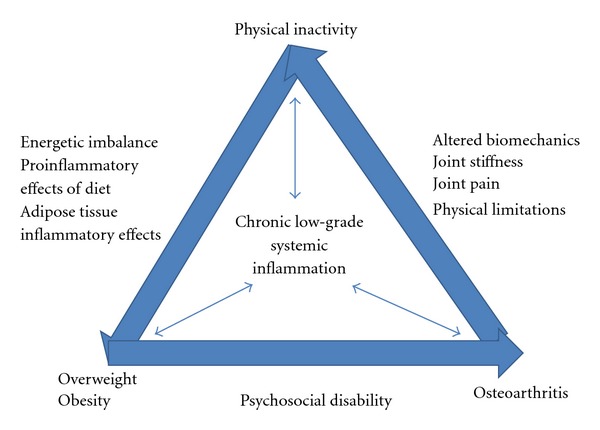
Relationships among osteoarthritis, obesity, and physical inactivity and relationship to the etiology of chronic low-grade systemic inflammation. Adapted from [8].

Figure 1 illustrates the interactive relationship among osteoarthritis, obesity, and physical inactivity. Obesity is an independent risk factor for osteoarthritis [84]. Although the mechanisms for this association are not completely understood, biomechanical loading and metabolic inflammation associated with excess adipose tissue and lipids may have a role. Pain associated with osteoarthritis leads to increasingly less activity and psychosocial and physical disability. Physical inactivity is an independent risk factor for inflammation due to the reduced expression of systemic and cellular anti-inflammatory mediators. Physiologic cyclic loading of cartilage tissue reduces the expression of proinflammatory mediators and decreases cytokine-induced extracellular matrix degradation. Physical inactivity reduces daily energy expenditure thereby promoting weight gain and continuation of the cycle. Emerging evidence indicates that osteoarthritis likely impedes the management of chronic metabolic conditions associated with prolonged negative lifestyle habits such as obesity, type 2 diabetes mellitus, and ischemic heart disease, because of its negative impact on physical activity.

Table 1 shows evidence for chronic low-grade inflammation and oxidative stress in people with osteoarthritis. Multiple comorbidities that share comparable underlying chronic low-grade inflammation and oxidative stress often coexist in individuals with chronic osteoarthritis, Examples of these conditions and synthesis of the evidence appear in Table 2, for example, atherosclerosis, chronic cancer, chronic obstructive lung disease, diabetes, hypertension, insulin resistance and metabolic syndrome, ischemic heart disease, obesity, and stroke. Almost 20 percent of American adults report having physician-diagnosed arthritis, and this is expected to increase over the next two decades [88]. Based on the Behavioral Risk Factor Surveillance System and National Health Interview Survey in the United States, individuals with osteoarthritis have a high incidence of other lifestyle-related conditions with inflammatory components that often present comorbidly with osteoarthritis (see examples in Table 2). Our search strategy used keywords including lifestyle-related conditions, chronic low-grade or chronic systemic inflammation. This synthesis of evidence reflects the literature indexed in established electronic data bases (MEDLINE and PubMed) and primarily published over the past five years. However, in several instances, important related work that was published earlier has been included in this evidence synthesis. The literature extracted represents a breadth of scholarly paradigms including clinical trials, cross-sectional population-based studies, experimental trials based on basic science and models and histological evidence, expert narrative reviews, randomized controlled clinical trials, and systematic reviews.

Although the degree to which the typical western lifestyle explains the prevalence of osteoarthritis is unclear, maximizing healthy living may have the greatest potential for minimizing its risk, its impact, and long-term outcomes including life-long health and wellbeing compared with invasive interventions including drugs and surgery and their related sequelae and side effects.

Overweight is now considered a leading condition associated with marked inflammation followed by arthritis, heart disease, and type 2 diabetes mellitus [89]. The mechanism whereby overweight contributes to inflammation is reported to involve high fat content of the diet [90]. Thus, promoting healthy weight through healthy nutrition in addition to regular physical activity and exercise is critically important to promote a maximally anti-inflammatory systemic environment to offset low-grade inflammation as well as to achieve weight loss.

## 4. Integration of Lifestyle Behavior Change into ‘‘First-Line” Management

For lifestyle behavior change to constitute ‘‘first-line” management as the literature would support, the health care team overall needs to share this goal and practice in partnership rather than in the conventional siloed care. The three primary health professions excluding, dentistry and pharmacy, include physicians, nurses, and physical therapists. Traditionally, physicians are highly trained in administration of invasive interventions, that is, drugs and surgery. Nurses have assumed a role in patient education over the years along with psychosocial considerations of patient care. Of the established health professions, physical therapy is the leading nonpharmacologic profession that is particularly well positioned to assume such an education role for patients related to healthy lifestyles and exercise [91, 92].

Consistent with the 21st century epidemiological trends, physical therapists are moving toward a model of care based on health (International Classification of Functioning, Disability and Health) [91, 93], which includes initiating and supporting behavior change such as optimal nutrition, weight reduction, reduced sedentary activity, and increased physical activity. With respect to nutrition, basic assessment can be done and education undertaken regarding patients' knowledge with the inflammatory characteristics of their diets and incorporating anti-inflammatory foods (see Table 3).

In addition, in the interest of best practice, as primary nonpharmacologic practitioners, contemporary physical therapists are integrating into practice health education including initiating and supporting smoking cessation, improved sleep hygiene, and stress management [94]. Given that smoking, poor sleep, and stress are all associated with low-grade inflammation and hyperimmune response, team members such as nutritionists and health counselors could be used to greater advantage on the health care team to promote effective health education related to health behavior change. In acute conditions, such education needs to be introduced potentially with pharmacologic intervention to reduce inflammation and pain expediently. However, as the acute episode subsides and the condition stabilizes, medication needs to be reduced as much as possible, and perhaps completely, as health living practices take maximal effect.

The benefits of healthy living have no better been exemplified than in an elegant but simple study reported by Ford and colleagues [95]. In their study of over 23,000 people between 35 and 65 years old, they reported that over an eight-year period, people who did not smoke; had a body mass index of less than 30 kg·m^2^; were physically active for at least 3.5 hours weekly; and ate healthily reduced their risk of type 2 diabetes mellitus by 93%, myocardial infarction by 81%, stroke by 50%, and cancer by 36%. Even if not all four health behaviors were present, risk of developing a chronic lifestyle-related condition decreased commensurate with an increase in the number of positive lifestyle factors. Furthermore, health-related quality of life increased with the number of healthy lifestyle behaviors that participants reported. In the process of conducting the present review of the literature, we identified no medication that was associated with such outcomes and such low risk of side effects, if any.

In the interest of best practice, healthy living recommendations need to be prescribed as uniquely for their direct effects on the pathoetiology of osteoarthritis, and prescribed as aggressively as first-line medications. Although general health recommendations are important for health promotion and disease prevention generally, the tenets of healthy living need to be systematically targeted to the patient's signs and symptoms and prescribed accordingly including long-term followup and support. Not doing so deprives the patient of evidence-informed best practice osteoarthritis management and care.

Consistent with healthy living as a first-line approach, patients' health behaviors need to be assessed in a measurable, reproducible, and standardized manner. In addition to questionnaires and self-reports, despite their limitations, inflammatory biomarkers such as C-reactive protein may be useful to objectively measure the effects of lifestyle behavior change rather than simply as an index of cardiovascular and diabetes risk [96–99].

To address the reports of health care practitioners about lack of knowledge and confidence to effect health behavior change, they have a range of evidence-based interventions at their disposal that are not time or resource intense [100–102]. In addition, the 5's approach of behavior change, for example, has some evidence base and has been endorsed by the World Health Organization [103]. Its simplicity makes it attractive to health professionals, that is, assess: evaluate behavior change status (and progress), advise: personally relevant behavioral recommendations, agree: set specific collaborative, feasible goals, assist: anticipate barriers, problem-solve solutions, and complete action plan, and arrange: schedule followup, contacts, and resources.

In the interest of best practice, lifestyle behaviors need to be systematically assessed in every patient and monitored across the health professions the patient is seeing. Healthy living recommendations need to be prescribed as uniquely for their direct effects on the pathoetiology of osteoarthritis as medications are, and as aggressively if first-line management is to truly reflect evidence-based practice. Although general recommendations are important for health promotion and disease prevention generally, healthy living recommendations must be systematically targeted to the patient's signs and symptoms. In addition to integrating dietary and activity recommendations, smoking cessation, sleep hygiene, and stress reduction should be included in the interest of comprehensive effective care. Not doing so deprives the patient of best practice osteoarthritis management in relation to potential comorbidities that commonly present in this cohort.

## 5. Implications: Clinical and Research

The evidence supporting lifestyle behaviour change to address low-grade inflammation in people with osteoarthritis often with coexistent lifestyle-related risk factors and low-grade inflammatory conditions (specifically, anti-inflammatory nutritional regimens, and moderate physical activity) is unequivocal. The evidence is sufficiently compelling for related healthy living assessment and recommendations be a component of first-line best practice in the management of the signs and symptoms of people with osteoarthritis. Assessments need to include lifestyle profiles related to body mass index, waist girth, and waist-to-hip ratio; physical activity and exercise, as well as smoking, sleep patterns, and stress (as these three latter factors have also been reported to be proinflammatory). When quantified in standardized ways, these profiles can serve as clinical outcomes to assess health behavior change interventions. The health behaviour change literature has exploded over the past two decades, yet health professions report lack of confidence in effecting health behavior change in their patients, and lack of resources including time [100]. Although much needs to be done, evidence-based interventions can be readily integrated into the framework of clinical practice and patient visits [101, 104], for example, brief advice, referral to others professionals, and followup). Physical therapists are particularly well positioned for initiating and supporting health behavior change in that patient visits tend to be prolonged and protracted over time, elements that are critical to effective long-term sustained health behavior change.

Studies are needed to examine the differentiating characteristics of those people with osteoarthritis who respond primarily to optimal nutrition and moderate physical activity, and those who do not. In addition, the elements of an anti-inflammatory nutrition regimen and moderate physical activity program need to be refined in terms of their prescriptive parameters, specifically, which elements should be a primary focus for which patients. Another line of studies is needed to examine the effect of such healthy lifestyle choices that increase inflammation threshold, on the need for medication and, if medication is indicated, how might its potency and dosage be reduced. The interactions among healthy lifestyle behaviors and pharmacokinetics need to be elucidated. Given that chronic systemic low-grade inflammation has been reported to be a common denominator of lifestyle-related conditions, studies are needed to establish the degree to which their risk factors and manifestations are reduced in people with chronic osteoarthritis whose first-line management includes prescribing optimal nutrition and physical activity for their anti-inflammatory effects. Furthermore, the impact of low-grade inflammation can be more far reaching than physical complaints alone, in that even healthy older adults report poorer health commensurate with level of inflammatory markers [105]. Lastly, all indicators support that the approach to chronic progressive conditions such as osteoarthritis needs to be holistic and interprofessional [106]. Research is needed to capture the breadth of this evidence-informed practice approach.

## 6. Conclusion

Based on the extant literature, exploitation of anti-inflammatory lifestyle behavior change as ‘‘first-line” intervention in the management of chronic osteoarthritis could well constitute best practice. Chronic low-grade inflammation that has been reported in chronic osteoarthritis is comparable to other lifestyle-related conditions supporting a common mechanism of action. Addressing chronic low-grade inflammation by focussing on lifestyle factors that contribute directly to it holds the promise of increasing a patient's inflammatory threshold, reducing rate of disease progression, reducing weight, and maximizing health by minimizing a patient's risk or manifestations of other lifestyle-related conditions. Even in part, such outcomes could minimize demands on physicians for short-term symptom reduction, and management of the patient's comorbidity related to lifestyle-related conditions. ‘‘First-line” lifestyle interventions to address chronic low-grade inflammation provides an informed cost-effective basis for the 21st century prevention, potential reversal, and management of chronic osteoarthritis. Exploitation of such ‘‘first-line” intervention, however, needs to be a goal shared and supported by all healthcare team members.

## Figures and Tables

**Table 1:** Synthesis of evidence of chronic low-grade inflammation being associated with osteoarthritis.

Authors	Title	Evidence classification	Methods	Findings	Conclusion
Cecil et al., 2005 The Journal of Immunology [9]	Inflammation-induced chondrocyte hypertrophy is driven by receptor for advanced glycation end products	Basic science study Experimental study	Analysis of human cartilage, cultured human articular chondrocytes, and recombinant human S100A11, soluble RAGE (advanced glycation end products), and RAGE-specific blocking antibodies	Normal human knee cartilages showed constitutive RAGE and S100A11 expression, and RAGE and S100A11 expression were upregulated in OA cartilages	Up-regulated chondrocyte expression in OA cartilage and RAGE signaling promote inflammation-associated chondrocyte hypertrophy
Rojas-Rodríguezet al., 2007 Medical Hypotheses [10]	The relation between the metabolic syndrome and energy-utilization deficit in the pathogenesis of obesity-induced osteoarthritis	Narrative review to examine a medical hypothesis: pathogenesis of obesity-induced OA may be explained by metabolic changes in striated muscle by interaction of insulin resistance and systemic inflammation in obese individuals	Evidence search strategy unspecified	Increased TH1 cytokines are produced by macrophages in presence of chronic infection and suppress insulin sensitivity Muscle cells and adipocytes are activated by inflammatory cytokines and contribute to chronic low-grade inflammation in apparently healthy obese individuals	The fatigue and muscle weakness induced by insulin resistance and inflammation in obese patients with metabolic syndrome (pro-inflammatory state) increase trauma to joints that result in breaking of tenoperiosteal junction and abrasive damage of cartilage
Schlesinger and Thiele, 2010 Annals of Rheumatic Diseases [11]	The pathogenesis of bone erosions are in gouty arthritis	Review	Synthesis of mechanical, pathological, cellular, and immunological factors role in the pathogenesis of bone erosions in gouty arthritis Search strategy unspecified	Monosodium urate crystal deposition associated with underlying OA Gouty tophus and bone erosions associated with chronic low-grade inflammation	Tophus eroding underlying bone is pivotal for development of bone erosions in gouty arthritis
Smith et al. 1997 Journal of Rheumatology [12]	Synovial membrane inflammation and cytokine production in patients with early osteoarthritis	Clinical trial of patients with varying stages of early OA (*n* = 63)	Synovial membrane samples obtained from the knees of patients	Thickening of lining layer, increased vascularity, and inflammatory cell infiltration in synovial membranes; changes proportional to severity Inflammatory markers increased in the synovial membranes of patients irrespective of degree of articular damage	Chronic inflammatory changes with production of pro-inflammatory cytokines characterize the synovial membranes of patients with early OA Low-grade synovitis results in the production of cytokines that may contribute to OA pathogenesis

**Table 2:** Synthesis of evidence of chronic low-grade inflammation being associated with conditions that may coexist with a diagnosis of osteoarthritis.

Alzheimer's disease					

Giunta et al., 2008 Journal of Neuroinflammation [42]	Inflammaging as a prodrome to Alzheimer's disease	Review The immunological aspects of aging related to Alzheimer's Disease (AD), that is, the increased innate immunity by cells of the mononuclear	Articles reported the characterization the aging immune response related to the concept of inflammaging (low-grade chronic up-regulation of pro-inflammatory response) Specific search strategy unspecified	Conditions of increased innate immune response with overproduction of pro-inflammatory proteins are related to healthy aging and AD People who age “well” have anti-inflammaging mechanisms and biomarkers that counter the adverse immune response of inflammaging	Countering inflammaging may prevent or treat the symptoms of AD
Veerhuis, 2011 Current Alzheimer Research [43]	Histological and direct evidence for the role of complement in the neuroinflammation of AD (AD-Alzheimer's disease)	Histologic and direct evidence	Synthesis and secretion of reactive oxygen species (ROS), cytokines, chemokines, and other potentially neurotoxic agents by the glial cells implicated in AD	In AD, there are brain areas with amyloid deposits and complement activation products Complement regulatory proteins are found in brain parenchyma and are upregulated, especially under inflammatory conditions	Evidence from immunohistochemical, in vitro and animal studies points to role for complement activation In chronic low-grade inflammatory conditions, such as in AD, complement activation proceeds, leading to sustained glial cell activation and neurodegenerative changes
Candore et al., 2010 Current Pharmacology Designs [44]	Low grade inflammation as a common pathogenetic denominator in age-related diseases: novel drug targets for anti-ageing strategies and successful ageing achievement	Review	Search strategy unspecified	Evidence supports that low-grade systemic inflammation characterizes ageing and that inflammatory markers are significant predictors of mortality with ageing	Elucidation of ageing pathophysiology to disentangle age-related low-grade inflammation will provide evidence to develop drugs to delay ageing process

Asthma					

Chou et al., 2011 Journal of Sex Medicine [45]	Asthma and risk of erectile dysfunction—a nationwide population-based survey	Population-based survey between 2000 and 2007, newly diagnosed asthma cases identified (18–55 y) (*n* = 3466) Control cohort (without asthma) matched for age and co-morbidities (*n* = 13,836)	Cohorts were followed for evidence of erectile dysfunction (ED)	Subjects with asthma experienced 1.9-fold increase in ED independent of age and comorbidity compared with control cohort	Asthma may be an independent risk factor for ED (increasing with asthma severity) Chronic systemic inflammation is implicated in this linkage
Dixon, 2012 Expert Reviews in Respiratory Medicine [46]	The treatment of asthma in obesity	Expert review	Epidemiology of obesity has influenced epidemiology of other conditions, for example, asthma Obesity major risk factor for new asthma Search strategy not specified	Mechanical factors, metabolic inflammation, and other comorbidities probably contribute to asthma	Therapies need to be developed and tailored to various underlying mechanisms
Juel et al., 2012 Journal of Asthma and Allergy [47]	Asthma and obesity: does weight loss improve asthma control? A systematic review	Systematic review	Obesity associated with high asthma incidence and poor control Review of knowledge on effect of weight reduction on asthma control based on systematic searches using the PubMed database and relevant MeSH terms	Weight loss in obese individuals associated with 48%–100% remission of asthma symptoms Weight loss associated with improved lung function and airway responsiveness to inhaled methacholine	Weight loss consistently reduces asthma symptoms Improved asthma control including objective measures of disease activity

Atherosclerosis					

Gu et al., 2012 Atherosclerosis [48]	Psychological stress, immune response, and atherosclerosis	Review	Synthesis of evidences that various immunological factors are transformed under prolonged psychological stress by causing vascular low-grade inflammation	Evidence supports expression of pro- and anti-inflammatory cytokines by stress hormones (catecholamines and corticosteroids)	Elucidation of two-way communication between neuroendocrine and immune systems Implications for targeted treatment strategies
Kucharz, 2012 Medical Hypotheses [49]	Chronic inflammation-enhanced atherosclerosis: can we consider it a new clinical syndrome?	Narrative review Medical hypothesis: incidence of cardiovascular disease (CVD) in patients with chronic autoimmune disorders is much higher than in general population CVD is caused by accelerated atherosclerosis, in which chronic inflammation is implicated	The literature search strategies unspecified	Chronic inflammation-enhanced atherosclerosis syndrome is proposed as a separate syndrome occurring in patients suffering of chronic inflammation	Atherosclerosis as an inflammatory disease and chronic extravascular inflammation have common mechanisms resulting in an increase in atherosclerosis and its sequelae, CVD
Lu et al., 2012 Psychosomatic Medicine [50]	Unpredictable chronic mild stress promotes atherosclerosis in high cholesterol-fed rabbits	Experimental	Chronic psychological stress associated increased with risk of atherosclerosis Study of effects of chronic stress on atherogenesis in rabbits Rabbits fed cholesterol-rich diet for 4–16 wks	High-cholesterol feeding resulted in hypercholesterolemia and formation of atherosclerotic plaques in the aorta High-cholesterol diet increased plaque size and instability	Findings support that atherosclerosis is augmented by chronic psychological stress, due to increased vascular inflammation and decreased endothelial nitric oxide bioavailability
Ortega et al., 2012 Atherosclerosis [51]	White blood cell count is associated with carotid and femoral atherosclerosis	Clinical study Subjects with dyslipidemia (*n* = 554) and sex-matched normolipidemic subjects (*n* = 246)	Examined the association between inflammatory markers and atherosclerosis evidence Carotid and femoral arteries were imaged White blood cell counts (WBCC) were obtained	Chronic low-grade inflammation is associated with atherosclerosis WBCC associated with measures of atherosclerosis independent of risk factors	WBCC is a useful and easy marker of atherosclerosis, consistent with its inflammatory basis
Pinto et al., 2012 Current Pharmaceutical Design [52]	Effects of physical exercise on inflammatory markers of atherosclerosis	Expert narrative review	Synthesis of research related to regular physical training and low-grade inflammation Search strategy unspecified	Physical exercise could be considered a useful weapon against local vascular and systemic inflammation in atherosclerosis.	Several mechanisms explain the positive effect of chronic exercise Including decreased inflammation and endothelial dysfunction, and modulated progression of underlying disease progress

Cancer					

Correa and Piazuelo, 2012 [53]	The gastric precancerous cascade	Lead article State-of-the-art	Review of experimental articles that support the steps in the gastric precancerous cascade Search strategy unspecified	Inflammatory changes may persist throughout the precancerous process First recognized histological change is active chronic inflammation which is the first step in the precancerous cascade	Most promising strategy for control of the condition is prevention, augmented by prolonging the pre-cancerous process which requires an understanding of the precancerous cascade Lesion detected earliest in inflammation
Peters et al. 2012 Stress [54]	Chronic psychosocial stress increases the risk for inflammation-related colon carcinogenesis in male mice	Experimental Animal model Investigated the effects of chronic psychosocial stress in male mice with artificially induced colorectal cancer (CRC)	Outcomes based on colonoscopic evaluation and protein analysis	CSC mice showed accelerated macroscopic lesions CSC mice showed more cell dysplasia than the single-housed control (SHC) mice Abnormal protein expression was also greater in CSC than SHC mice	Findings consistent with the fact that chronic psychosocial stress increases the likelihood of developing an irritable bowel, and multiple types of malignant neoplasms, including CRC

Chronic obstructive lung disease					

Cox jr 2012 Dose Response [55]	Dose-response thresholds for progressive diseases	Narrative review To provide evidence base for framework	Framework proposed for understanding how exposure can destabilize normally homeostatic feedback control systems and create sustained imbalances and elevated levels of disease-related	The resulting model, called the alternative equilibria (AE) theory, implies the existence of an exposure threshold below which transition to the alternative equilibrium (potential disease)	These predictions may help to explain patterns observed in experimental and epidemiological data for diseases such as COPD, silicosis, and inflammation-mediated lung cancer
			variables, by creating a new, locally stable, alternative equilibrium for the dynamic system, in addition to its normal (homeostatic) equilibrium Search strategy unspecified	state will not occur, and once exceeded, progression to the alternative equilibrium continues spontaneously, even without further exposure	
Lindberg et al., 2011 COPD [56]	Co-morbidity in mild-to-moderate COPD: comparison to normal and restrictive lung function	Clinical trial Subjects with COPD from obstructive lung disease in northern Sweden cohort followed in 2002–2004 (*n* = 993) Gender and age matched reference subjects without COPD (*n* = 993)	To evaluate if conditions associated with systemic inflammation (e.g., cardiovascular diseases, diabetes, chronic rhinitis, and gastroesophageal reflux, are overrepresented in patients with COPD Analysis based on interview data on co-morbidity and symptoms	Prevalence of chronic rhinitis and gastroesophageal reflux (GERD) was higher in COPD compared to reference group In restrictive lung function, the prevalence of chronic rhinitis, cardiovascular disease, hyperlipemia, and diabetes was higher compared to reference group In COPD and heart disease, chronic rhinitis and/or GERD were proportionately higher than reference group	Co-morbid conditions associated with systemic inflammation, for example, cardiovascular disease, chronic rhinitis, and gastroesophageal reflux, were common in patients with COPD Overlap between heart disease, chronic rhinitis and GERD was large in COPD
ten Hacken, 2009 Proceeding of the American Thoracic Society [57]	Physical inactivity and obesity: relation to asthma and chronic obstructive pulmonary disease?	Review To summarize the available literature regarding the potential role of physical inactivity and obesity in asthma and COPD and to examine their contribution to systemic inflammation	Physical inactivity and obesity are associated with low-grade systemic inflammation that may contribute to the inflammatory processes present in many chronic diseases Search strategy unspecified	High prevalence of asthma in obesity In chronic obstructive pulmonary disease (COPD), physical inactivity has been demonstrated This was associated with a higher degree of systemic inflammation,	Elucidation of the independent relationship between physical inactivity and obesity with systemic inflammation, performance-based studies of physical inactivity in asthma and COPD are needed
				independent of body mass index Obesity is associated with the chronic obstructive phenotype and features of the metabolic syndrome	
Wouters et al., 2009 Proceedings of the American Thoracic Society [58]	Systemic and local inflammation in asthma and chronic obstructive pulmonary disease: is there a connection?	Review State-of-the-art	To examine the association between asthma and chronic obstructive pulmonary disease (COPD) Search strategy unspecified	Spillover of inflammatory mediators into the circulation considered the source of systemic inflammation in these conditions	Nature of systemic inflammation remains unclear Adipose tissue mediated inflammation is one explanation

Diabetes mellitus (types 1 and 2)					

Chang et al., 2012 in press Acta Diabetologica [59]	Acute and chronic fluctuations in blood glucose levels can increase oxidative stress in type 2 diabetes mellitus	Clinical trial Subjects: patients with type 2 diabetes mellitus (*n* = 34)	To examine whether short- or long-term glycemic fluctuations could induce oxidative stress and chronic inflammation, relationships between glycemic variability, oxidative stress markers, and high-sensitivity C-reactive protein (hs-CRP) were studied	Relationships between markers for short- and long-term glycemic control remained significant with respect to oxidative stress and chronic inflammation, after adjusting for other markers of diabetic control	Both acute and chronic blood glucose variability can induce oxidative stress and chronic inflammation
van Bussel et al., 2012 in pressNutrition and Metabolism in Cardiovascular Disease [60]	Unhealthy dietary patterns associated with inflammation and endothelial dysfunction in type 1 diabetes: The EURODIAB study	Clinical trial To investigate the association between nutrient consumption and biomarkers of endothelial dysfunction (ED) and low-grade inflammation (LGI) in subjects with type 1 diabetes (*n* = 491)	A healthy diet has been inversely associated with ED and LGI Nutrient consumption and lifestyle risk factors were measured in 1989 and 1997 Biomarkers of ED and LGI (C-reactive protein, interleukin 6, and tumour necrosis factor *α*) were measured in	Consumption of less fibre, polyunsaturated fat and vegetable protein, and more cholesterol over the study period was associated with more ED and LGI	Following dietary guidelines in type 1 diabetes may reduce cardiovascular disease risk by favourably affecting ED and LGI
			1997 and averaged into *Z*-scores. The nutrient residual method was used to adjust individual nutrient intake for energy intake		

Fibromyalgia					

Kadetoff et al., 2012 Journal of Neuroimmunology [61]	Evidence of central inflammation in fibromyalgia-increased cerebrospinal fluid interleukin-8 levels	Clinical trial Subjects: patients with FM	To assess intrathecal concentrations of pro-inflammatory substances in patients with FM	Elevated cerebrospinal fluid and serum concentrations of interleukin-8, but not interleukin-1beta, in FM patients	Findings consistent with a central pro-inflammatory component
Ortega et al. 2012 Journal of Medical Science and Sports [62]	Aquatic exercise improves the monocyte pro- and anti-inflammatory cytokine production balance in patients with fibromyalgia (FM)	Clinical trial Subjects: women patients with FM and age-matched control group of healthy women	Evaluated the effect of a pool-aquatic exercise program (8 months, two weekly 60 min sessions) on the inflammatory cytokine production by isolated monocytes, and on the serum concentration of C-reactive protein (CRP)	Monocytes from FM patients released more inflammatory cytokines than those from women in control group FM women had high circulating concentrations of CRP Increased IL-6 with a concomitant decreased TNF*α* spontaneous release was found after 4 months Anti-inflammatory effect of the exercise program was also corroborated by a decrease in the circulating CRP concentration	FM is associated with chronic inflammation that can be offset with physical exercise such as aquatic exercise Exercise also improved the health-related quality of life of the FM patients

Hypertension					

Berni et al., 2012 Journal of Human Hypertension [63]	Renal resistive index and low-grade inflammation in patients with essential hypertension	Clinical trial Subjects: hypertensive patients (*n* = 85; 57 ± 14 years, 61 males) without diabetes, renal	To study the relationship between RRI and serum hsCRP in hypertensives with preserved renal function, without	Patients with pathologic RRI (*n* = 21) were older and had higher hsCRP levels compared with patients with normal RRI, as well as patients	HsCRP is a predictor of both pathologic RRI and decreased RV/RRI, even after adjustment In essential hypertension,
		failure, microalbuminuria, or major inflammatory disease	microalbuminuria	with decreased RV/RRI (*n* = 43) HsCRP was directly related with RRI and inversely with RV/RRI	low-grade inflammation is associated with tubulointerstitial damage
He et al., 2012 Journal of Hypertension [64]	Metformin-based treatment for obesity-related hypertension: a randomized, double-blind, placebo-controlled trial	Randomized, double-blind, placebo-controlled trial Subjects: participants randomized to metformin (*n* = 180) and participants randomized to placebo (*n* = 180)	To explore whether metformin-based treatment (which reduces weight and inflammation in diabetes) benefits obesity-related hypertension without diabetes 24 week drug trial	Metformin compared with placebo did not have effects on blood pressure, blood glucose, and high-density or low-density lipoprotein cholesterol, but it did reduce total serum cholesterol Metformin reduced weight, BMI, waist circumference and both subcutaneous and visceral adiposity and lowered serum high-sensitivity C-reactive protein	Results supported an inflammatory component of hypertension in patient who are obese, that was amenable to metformin that targets inflammation
Sari et al. 2011 Clinical Experimental Hypetension [65]	The effect of quinapril treatment on insulin resistance, leptin and high sensitive C-reactive protein in hypertensive patients	Clinical trial Subjects: hypertensive patients (*n* = 54) and control subjects (*n* = 24)	To evaluate the effect of quinapril on HOMA-IR, high sensitive C-reactive protein, and leptin Blood pressure, leptin, high sensitive C-reactive protein, and HOMA-IR were determined at baseline and after 3 months of quinapril treatment	After treatment with quinapril HOMA-IR, high sensitive C-reactive protein, and leptin were decreased in hypertensive patients	Quinapril may be used as a therapy for improving blood pressure as well as the insulin resistant, hyperleptinemic, and low-grade inflammatory state in hypertension
Sugiura et al. 2011 Journal of Clinical Lipidology [66]	Impact of lipid profile and high blood pressure on endothelial damage	Clinical trial Japanese male outpatients with grade I or II hypertension,	Blood was sampled for laboratory analysis and endothelial	Total cholesterol to high-density lipoprotein cholesterol ratio	Impaired endothelial function was associated with increased
		along with gender and age-matched normotensive subjects (both *n* = 25)	function was assessed by flow-mediated dilation (FMD)	(total-C/HDL-C) was inversely correlated with the FMD value and positively correlated with both malondialdehyde-modified low-density lipoprotein and high-sensitivity C-reactive protein values to those in normotensive subjects with high total-C/HDL-C	total-C/HDL-C values, possibly as a result of increased vascular oxidative stress and inflammation In early stages of atherosclerosis, the impact of both total-C/HDL-C and BP may be similar in terms of endothelial damage

Insulin resistance/metabolic syndrome					

Piya et al., 2006 in press Journal of Endocrinology [67]	Adipokine inflammation and insulin resistance: the role of glucose, lipids and endotoxin	Review	To examine impact of nutrients such as glucose and lipids on inflammatory pathways, specifically within adipose tissue, and how these influence adipokine inflammation and insulin resistance Search strategy unspecified	Through overnutrition, glucose, lipids, and endotoxin affect different tissues to mediate an aberrant inflammatory response and augment pathogenesis of insulin resistance and metabolic disease	Evidence supports the persistent insults from dysfunctional diets that need to be the targets of intervention Reducing the burden in this way may impact people's long-term health
Shoelson et al., 2006 Journal of Clinical Investigation [68]	Inflammation and insulin resistance	Review	Evidence has linked inflammation to the pathogenesis of type 2 diabetes (T2D) Search strategy unspecified	With discovery of an important role for tissue macrophages, these findings are helping to reshape thinking about how obesity increases the risk for T2D and metabolic syndrome	The evolving concept of insulin resistance and T2D as having immunological components and as improving the picture of how inflammation modulates metabolism provides new opportunities for using anti-inflammatory strategies to address metabolic consequences of excess adiposity

Ischemic heart disease					

Simon, 2012 Circulation Journal [69]	Inflammation and vascular injury	Review	To examine the central role of inflammation in vascular injury and repair Search strategy unspecified	Binding site for GPIb**α**in Mac-1 shows that leukocyte engagement of platelet GPIb**α**via Mac-1is critical for the biological response to vascular injury, thrombosis, vasculitis, glomerulonephritis, and multiple sclerosis	Almost all inflammation is platelet dependent Ligand engagement of Mac-1 initiates a novel gene that promotes inflammation
Kalogeropoulos et al., 2012 Heart Failure Clinics [70]	From risk factors to structural heart disease: the role of inflammation	Review	Review strategy unspecified	Elevated levels of circulating proinflammatory cytokines and adipokines have been repeatedly associated with increased risk for clinically manifest (Stage C) heart failure in large cohort studies. The role of low-grade, subclinical inflammatory activity in the transition from risk factors (Stage A heart failure) to structural heart disease (Stage B heart failure) is less well understood	Recent evidence suggests that chronic low-grade inflammatory activity is involved in most mechanisms underlying progression of structural heart disease, including ventricular remodeling after ischemic injury, response to pressure and volume overload, and myocardial fibrosis Inflammation also contributes to progression of peripheral vascular changes
Vizzardi et al. 2011 Panminerva Medica [71]	*Helicobactor pylori* and ischemic heart disease	Review	Many studies have been performed on the relationship between infection from *Helicobacter pylori* and atherosclerotic diseases, like stroke and ischemic heart disease	Review of the literature that has investigated the role of HP in the development and pathogenesis of CAD. Infection could lead to IHD through pathways such as endothelial cells	Results from these studies have raised new perspectives on coronary heart disease, especially regarding the possibility of modifying the clinical history of the disease through eradication of these
			Some infections could have a role on the genesis and development of damage to the vascular wall and of atheromatous plaque HP could influence the development of IHD through various pathways Search strategy unspecified	colonization, changes in the lipid profiles, increased coagulation and platelet aggregation levels, induction of molecular mimicry mechanisms, and the promotion of a low-grade systemic inflammation	infective microorganisms Further studies indicated

Kidney disease					

Kang et al., 2012 Journal of Korean Medical Science [72]	Low-grade inflammation, metabolic syndrome and the risk of chronic kidney disease: a 2005 Korean National Health and Nutrition Examination Survey	Cross-sectional study Subjects: adults registered in the national survey (*n* = 5291)	To examine the relationship between white blood cell (WBC) count and chronic kidney disease ≥stage 3 Measures of glomerular filtration rates	Low-grade inflammation is associated with chronic kidney disease in people with metabolic syndrome ≥stage 3	Low-grade inflammation associated with chronic kidney disease ≥stage 3 in people with metabolic syndrome suggests new treatment approaches
Kocyigit et al., 2012 American Journal of Nephrology [73]	Early arterial stiffness and inflammatory bio-markers in normotensive polycystic kidney disease patients	Clinical trial Cross-sectional design Patients (*n* = 50) with autosomal-dominant kidney disease (ADPKD) (42% males, 36.6 ± 9.9 years, no blood pressure medication) and healthy controls (*n* = 50) (44% males, 35.4 ± 6.4 years)	To clarify temporal relationship between ADPKD, hypertension, and the loss of renal function, patients with early-stage ADPKD who did not yet have hypertension were examined Pulse wave velocity (PWV), cardiac morphology and function, aortic elastic indexes, estimated glomerular filtration rate (eGFR), 24-hour ambulatory blood pressure, interleukin-6 (IL-6), tumor necrosis factor-*α* (TNF-*α*), and highly sensitive C-reactive protein (hs-CRP) were measured	Despite normal blood pressure, aortic stiffness index and pulse wave velocity values were increased in patients compared to controls In univariate analysis, IL-6, TNF-*α*, hs-CRP, and eGFR were correlated with PWV PWV is predicted by IL-6, TNF-*α*, and hs-CRP	Increased arterial stiffness and pulse wave velocity are early manifestations of ADPKD appearing before hypertension or reduced eGFR These vascular abnormalities are related to signs of systemic low grade inflammation Findings support a common pathophysiological mechanism apparently present also in other vascular diseases
Luis- Rodríguez et al., 2012 World Journal of Diabetes [74]	Pathophysiological role and therapeutic implications of inflammation in diabetic nephropathy	Review (experimental and clinical studies)	To identify pathogenic pathways for earlier diagnosis and targeting novel treatments Search strategy unspecified	Activation of innate immunity with development of a chronic low grade inflammatory response is a recognized factor in the pathogenesis of diabetic nephropathy Experimental and clinical studies support various inflammatory molecules and pathways in the pathoetiology of diabetic neuropathy	Increased knowledge and understanding of inflammatory mechanisms are needed to augment clinical interventions for this complication
Tang et al., 2012 International Journal of Nephrology [75]	Inflammation and oxidative stress in obesity-related glomerulopathy	Review	To focus on inflammation and oxidative stress in the progression of obesity-related glomerulopathy and possible interventions to prevent kidney injury in obesity Search strategy unspecified	Obesity-related glomerulopathy is a major cause of end-stage renal disease. Obesity has been considered a state of chronic low-grade systemic inflammation and chronic oxidative stress Augmented inflammation in adipose and kidney tissues promotes the progression of kidney damage in obesity	Adipose tissue, which is accumulated in obesity, is a key endocrine organ that produces multiple biologically active molecules, including leptin, adiponectin, and resistin, that affect inflammation Oxidative stress is also associated with obesity-related renal diseases and may trigger the initiation or progression of renal damage in obesity Both inflammation and oxidative stress induce damage to renal tubule and glomerulus and result in endothelial dysfunction in the kidney Anti-inflammation and antioxidant
					interventions may be therapies to prevent and treat obesity-related renal diseases

Obesity					

Hulsmans et al., 2012 PLoS One [76]	Interleukin-1 receptor-associated kinase-3 is a key inhibitor of inflammation in obesity and metabolic syndrome	Experimental and clinical studies Obese individuals (*n* = 21 and 102) and age-matched controls (*n* = 46)	Cluster of molecules were studied that support interactions between the stress conditions of low-grade inflammation and oxidative stress in monocytes Effect of three month weight loss after bariatric surgery examined	Visceral obesity is associated with type 2 diabetes and metabolic syndrome Low-grade chronic inflammation and oxidative stress synergize in obesity and obesity-induced disorders Odds ratio of high-sensitivity C-reactive protein, a widely used marker of systemic inflammation, was 4.3	Weight loss was with a lowering of systemic inflammation and a decreasing number of metabolic syndrome components An increase in reactive oxygen species in combination with obesity-associated low adiponectin and high glucose and interleukin-6 was identified as the cause of the decrease in IRAK3 in THP-1 cells in vitro
Issa and Griffin, 2012 Pathobiology of Aging and Age Related Diseases [8]	Pathobiology of obesity and osteoarthritis: integrating biomechanics and inflammation	Review	Search strategy unspecified	Pathobiology of obesity and osteoarthritis (OA) was examined, as well as literature the underlying systemic inflammation, its relationship to inactivity, and their interactions	Inflammation is central to progression of the disease cycle involving obesity, osteoarthritis, and physical inactivity Metabolic inflammation is believed to contribute to metabolic inflexibility and on-going production of pro-inflammatory mediators Findings support that metabolic inflammation increases OA risk
Rico-Rosillo and Vega-Robledo, 2012 Revista Médica del Instituto Mexicano del Seguro Social [77]	New trends in macrophages, inflammation and adipose tissue	Review	To highlight the macrophage participation in the generation of obesity-induced inflammation Search strategy unspecified	Accumulating evidence suggest the involvement of adipose tissue derived proteins, collectively known as adipokines as well as other factors produced in this tissue by cells besides adipocytes, like fibroblasts, lymphocytes, and macrophages Obesity burden on health extends across multiple organs systems and diseases (atherosclerosis, coronary heart diseases, osteoarthritis, diabetes, hypertension, and dyslipidemia)	Obesity is considered a low-inflammatory condition An increasing number of reports suggest that the adipose tissue itself might be a source of pro-inflammatory factors and a target of inflammatory processes Evidence supports involvement of adipose tissue-derived proteins, collectively known as adipokines and other factors produced in this tissue by cells besides adipocytes (fibroblasts, lymphocytes, and macrophages)
Stienstra, 2007 PPAR Research [78]	PPARs, obesity, and inflammation	Review	To address the role of peroxisome proliferator-activator receptors (PPARs) in obesity-induced inflammation specifically in adipose tissue, liver, and the vascular wall Search strategy unspecified	Changes in inflammatory status of adipose tissue and liver with obesity supports co-existent chronic low-level inflammation Various molecular mechanisms have been implicated in obesity-induced inflammation (some modulated by PPARs) PPARs modulate the inflammatory response, hence, constitute a therapeutic target to mitigate obesity-induced inflammation and its consequences	Obesity is accompanied with fat storage in tissues other than adipose tissue (liver and skeletal muscle) which may lead to local insulin resistance and stimulate inflammation Obesity changes the morphology and composition of adipose tissue, leading to changes in its protein production and secretion including pro-inflammatory mediators
Tajik et al. 2012 in press Journal of Endocrinological Investigation [79]	Effect of diet-induced weight loss on inflammatory cytokines in obese women	Clinical trial Subjects: Premenopausal obese women (body mass index ≥ 30) aged 21 to 54 years without diabetes, hypertension, or hyperlipidemia (*n* = 29)	To evaluate changes in pro/anti-inflammatory adipocytokines and metabolic profile after moderate diet-induced weight, anthropometric parameters, lipid and glucose profiles, IL-6, IL-10, and IL-18 were measured Subjects then entered into a weight reduction program (3 months)	Body mass index, waist circumference, triceps skinfold thickness, total cholesterol, triglyceride, and fasting plasma glucose decreased, while HDL-cholesterol increased While plasma levels of IL-6 and IL-18 decreased, no change was observed in circulating levels of IL-10	Obesity is associated with low-grade systemic inflammation which has been linked to the increased risk of cardiovascular disease and type II diabetes in obese patients improved body composition induced by restriction of energy intake is associated with favorable serum concentrations of IL-6 and IL-18 in obese women

Rheumatoid arthritis					

Gremese and Ferraccioli 2011 Autoimmunology Review [80]	The metabolic syndrome: the crossroads between rheumatoid arthritis and cardiovascular risk	Review	Rheumatoid arthritis (RA) patients have an incidence of cardiovascular (CV) diseases two-fold that of the general population Atherosclerosis, the main determinant of CV morbidity and mortality, and carotid intima-media thickness, an early preclinical marker of atherosclerosis, also occur early on in RA Search strategy unspecified	CV risk factors seem to have the same prevalence in RA and non-RA patients, thus they do not fully explain increased CV burden, suggesting that RA inflammation and therapies play a role in increasing CV risk in these patients The metabolic syndrome (MetS) and fat tissue are likely major players in this complex network The association of MetS and atherosclerosis is partly mediated by altered secretion of adipokines by adipose tissue and,	Obesity is now regarded as a systemic, low-grade inflammatory state, and inflammation as a link between obesity, metabolic syndrome, and CV diseases To control CV risk, data support the necessity of “tight control” of inflammation from both RA and MetS
				on the other hand, there are evidence that adipokines may play a role in inflammatory RA	
Prete et al., 2011 Autoimmunology Review [81]	Extra-articular manifestations of rheumatoid arthritis: An update	Review Rheumatoid arthritis (RA) is an immune-mediated disease involving chronic low-grade inflammation that may progressively lead to joint destruction, deformity, disability, and even death Despite its predominant osteoarticular and periarticular manifestations, RA is a systemic disease often associated with cutaneous and organ-specific extra-articular manifestations (EAM)	Current Reviews knowledge about EAM in terms of frequency, clinical aspects, and current therapeutic approaches. In an initial attempt at a classification, we separated EAM from RA co-morbidities and from general, constitutional manifestations of systemic inflammation. EAM was classified as cutaneous and visceral forms, both severe and not severe Search strategy unspecified	In aggregated data from 12 large RA cohorts, patients with EAM, especially the severe forms, were found to have greater co-morbidity and mortality than patients without EAM	Understanding the complexity of EAM and their management remains a challenge for clinicians, especially since the effectiveness of drug therapy on EAM awaits study

Stroke					

Denes et al. 2011 Cerebrovascular Disease [82]	Interleukin-1 and stroke: biomarker, harbinger of damage, and therapeutic target	Review	Inflammation is established as a contributor to cerebrovascular disease Risk factors for stroke include many conditions associated with chronic or acute inflammation, and inflammatory changes in the brain after cerebrovascular events contribute	Evidence supports importance of peripherally-derived immune cells and inflammatory molecules in various central nervous system disorders, including stroke Inflammatory cytokine, interleukin-1 (IL-1), plays a pivotal role in both local and systemic	Blockade of IL-1 could be therapeutically useful in several diseases which are risk factors for stroke There is considerable preclinical and clinical evidence that inhibition of IL-1 by IL-1 receptor antagonist may be valuable in the management of acute stroke
			to outcome in experimental studies, with growing evidence from clinical research Search strategy unspecified	inflammation and is a key driver of peripheral and central immune responses to infection or injury	
Wu et al., 2012 American Journal of Rhinological Allergy [83]	Risk of stroke among patients with rhinosinusitis: a population-based study in Taiwan	Population-based trial Prospective cohort study Patients in Taiwan (Longitudinal Health Insurance Database 2005 (LHID2005)) who had received a diagnosis of rhinosinusitis (*n* = 53,653) between January 1, 2004 and December 31, 2005 Control group (1 : 4) drawn from the same database was matched for age and gender (*n* = 214,624)	Each patient was followed up using data entered until the end of 2006 Proportional hazard regressions were performed to evaluate the hazard ratios (HRs) after adjusting for potential confounding factors	Patients with rhinosinusitis were more likely to suffer strokes than the control population, after adjusting for potential confounders	Both acute and chronic sinusitis are risk factors or markers for stroke that is independent of traditional stroke risk factors Further epidemiological research is warranted

**Table 3:** Pro- and Anti-inflammatory Foods (Source: reviews Beck, 2010; Daniluk, 2011; Weil, 2012; [85–87]).

Pro-inflammatory Foods	Anti-inflammatory Foods
Alcohol	The “anti-inflammatory” nutritional plan includes:
Regular high consumption irritates esophagus, larynx and liver which can lead to chronic inflammation which promotes tumor growth at sites of chronic irritation	Avoidance of sweets and sugar
	Avoidance of high refined foods such as processed foods (white bread and rice, and pasta), Minimal fats (virgin olive oil okay as it has excellent anti-inflammatory properties) High fiber foods including dark breads such as rye and pumpernickel No alcohol
Cooking oils	Recommended anti-inflammatory foods:
A diet of high imbalance of omega-6 to omega-3 ratio promotes inflammation (e.g., heart disease and cancer)	Oatmeal (not instant) Asparagus, avocado, beets, Brussell sprouts, broccoli, caulflower, kale, parsnip, spinach Romaine lettuce Berries Strawberries, blueberries, raspberries, blackberries Green apples, oranges, pears, lemons, cantaloupe melon Olives Unsalted raw nuts Sunflower seeds Extra virgin olive oil Water Green tea Beans, chickpeas, black beans Lentils Low fat turkey/chicken Eggs Salmon Low sodium tuna packed in water Dairy Low-fat milk products are acceptable particularly plain yogurt, cottage and solid cheeses, if any, like Swiss or cheddar; feta
Dairy products	
Meat (commercially produced meats where animals are fed grains such as soy beans and corns (a diet high in inflammatory omega-6 fatty acids and low in anti-inflammatory omega-3 fatty acids; also, these animals have limited exercise and raised to gain excess fat, ending up with high saturated fats. To make the animals grow faster and prevent them from getting sick, they are injected with hormones and fed antibiotics.) Red meats (beef, lamb and pork) and processed meats (has, sausages and salami)	
Red meat contains a molecule humans do not naturally produce (Neu5Gc) that leads to the production of antibodies in defense of it, an immune response that may trigger chronic inflammation, and low grade inflammation (linked to heart disease and cancer)	
Refined grains	
Devoid of fiber and vitamin B compared with unrefined grains (have bran, germ and aleurone layer); refined grains like refined sugar with high glycemic index When consistently consumed hasten onset heart disease and cancer Also often laden with fat and sugar and artificial flavors and partially hydrogenated oil	
Artificial food additives	
Aspartame and monosodium glutamate reportedly trigger inflammatory responses (particularly in those with inflammatory conditions, for example, rheumatoid arthritis	
Sugars	
Trans fats (found in deep fried foods, commercially baked goods, and those prepared with partially dehydrongenated oil, margarine and vegetable shortening	
